# A chloroplast genomic strategy for designing taxon specific DNA mini-barcodes: a case study on ginsengs

**DOI:** 10.1186/s12863-014-0138-z

**Published:** 2014-12-20

**Authors:** Wenpan Dong, Han Liu, Chao Xu, Yunjuan Zuo, Zhongjian Chen, Shiliang Zhou

**Affiliations:** State Key Laboratory of Systematic and Evolutionary Botany, Institute of Botany, the Chinese Academy of Sciences, Beijing, 100093 China; Institute of Sanqi Research, Wenshan College, Wenshan, 663000 Yunnan China; Shanghai Chenshan Plant Science Research Center, the Chinese Academy of Sciences, Shanghai Chenshan Botanical Garden, Shanghai, 201602 China

**Keywords:** *Panax*, Chloroplast genome, DNA mini-barcode, *ycf1*

## Abstract

**Background:**

Universal conventional DNA barcodes will become more and more popular in biological material identifications. However, in many cases such as processed medicines or canned food, the universal conventional barcodes are unnecessary and/or inapplicable due to DNA degradation. DNA mini-barcode is a solution for such specific purposes. Here we exemplify how to develop the best mini-barcodes for specific taxa using the ginseng genus (*Panax*) as an example.

**Results:**

The chloroplast genome of *P. notoginseng* was sequenced. The genome was compared with that of *P. ginseng*. Regions of the highest variability were sought out. The shortest lengths which had the same discrimination powers of conventional lengths were considered the best mini-barcodes. The results showed that the chloroplast genome of *P. notoginseng* is 156,387 bp. There are only 464 (0.30%) substitutions between the two genomes. The intron of *rps16* and two regions of the coding gene *ycf1, ycf1a* and *ycf1b*, evolved the quickest and served as candidate regions. The mini-barcodes of *Panax* turned out to be 60 bp for *ycf1a* at a discrimination power of 91.67%, 100 bp for *ycf1b* at 100%, and 280 bp for *rps16* at 83.33%.

**Conclusions:**

The strategy by searching the whole chloroplast genomes, identifying the most variable regions, shortening the focal regions for mini-barcodes are believed to be efficient in developing taxon-specific DNA mini-barcodes. The best DNA mini-barcodes are guaranteed to be found following this strategy.

**Electronic supplementary material:**

The online version of this article (doi:10.1186/s12863-014-0138-z) contains supplementary material, which is available to authorized users.

## Background

DNA barcoding is a relatively new concept, aiming to provide rapid, accurate and automatable species identification using a standard DNA region. Chloroplast (or plastid) sequences such as *rbcL* and *matK* are usually used as DNA barcodes for plant [1]. The lengths of the commonly used barcoding markers are longer than 650 bp. In most cases it is easy to achieve PCR success when using DNA of high quality. However, if the DNA molecules have degraded into fragments shorter than the spanning length of the primers, say 650 bp, it would not be possible to amplify the DNA barcodes. In these cases, DNA mini-barcodes could be used.

A DNA mini-barcode is a short DNA, generally 100–250 bp [2], suitable for species identification. Thus far, a few tries have been made to design DNA mini-barcodes [3,4]. Owing to significantly reduced length of sequences, PCR amplification success should presumably be much improved, but identification success would thus be hampered. A good DNA mini-barcode should be of high PCR and sequencing successes without much lowering species discrimination power. Therefore, DNA mini-barcodes are more often taxon specific than universal. Preferably DNA mini-barcodes should be the most informative regions of a genome. For seed plants, it is now realistic to find such DNA mini-barcodes by searching the whole chloroplast genomes owing to the ease of chloroplast genome sequencing [5].

Chloroplast sequences have been extensively used for species identification and phylogenetic reconstruction of plants. Chloroplast sequences evolve relatively slowly and there are not very many substitutions between species within a genus. To find the best DNA mini-barcodes, whole chloroplast genome screening is usually necessary. Typically, the chloroplast genome size of higher plants ranges from 120 to 160 kb, and a pair of inverted repeats (IRs) divides the genome into a large single copy (LSC) region and a small single copy (SSC) region. The IR regions are quite conservative [6], and the variable regions locate predominantly in the LSC and SSC [7].

DNA mini-barcodes can be used for species identification of digested material [8], old herbarium/museum specimens [9], ancient DNA, and more frequently processed medicinal herbs when high-quality DNA is not available and degraded DNA has to be used. Ginsengs (*Panax spp., Araliaceae*) are the best known Chinese medicine worldwide. They have been used as medicines alone or in combinations with other medicines. Recently, ginsengs were also used as an ingredient of cosmetics, tooth paste, beverage, vegetable, etc. There are eight species in *Panax*. All species are considered seriously endangered medicinal plants. *Panax notoginseng* (Burkill) F. H. Chen ex C. Y. Wu & K. M. Feng is extinct in the wild and wild *P. ginseng* C.A. Mey. in China is nearly extinct. However, illegal harvest and trade happen occasionally. For law-enforcement activities in conservation of wild populations of endangered species, there is a need for a method for correct identification of confiscated materials in forms of fragments, powders or decoctions of any organs. *Panax ginseng* and *P. notoginseng* have been cultivated in China for a long time for medicinal purposes. Roots of *P. quinquefolius* L. are imported from the USA or produced in the Northeast China. The commercial roots of *P. ginseng* and *P. quinquefolius* resemble each other and it is difficult for laymen to tell them apart. When they were sliced or powdered, it is unlikely for experts to distinguish them. Almost all species are identifiable using the DNA barcoding method according to Zuo et al. [10]. However, if the materials were processed as in decoctions and dietary supplements, the conventional DNA barcodes would probably fail. Therefore, it is justified to design DNA mini-barcodes of ginsengs for conservation purpose and for monitoring ginseng market and protecting consumers’ rights.

In this study, we report a strategy of designing taxon-specific DNA mini-barcodes using ginsengs as an example. We first sequenced the chloroplast genome of *P. notoginseng*, then we sought out the hypervariable regions by comparing the new genome to the one of *P. ginseng*, and finally we determined the length and positions of the best DNA mini-barcodes and tested their applicability.

## Results

### Characteristics of the chloroplast genome of *P. notoginseng*

**Figure 1:**
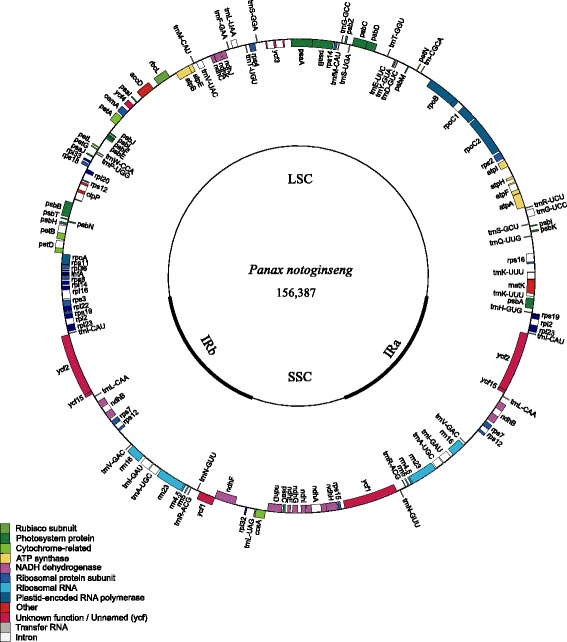
**Representative map of the chloroplast genome of** ***Panax notoginseng.*** The annotation of the genome was performed using DOGMA. The genes that are drawn outside of the circle are transcribed clockwise, while those inside are counterclockwise. Small single copy (SSC), large single copy (LSC), and inverted repeats (IRa, IRb) are indicated.

The chloroplast genome of *P. notoginseng* is 156,387 bp in length, slightly longer than the genome of *P. ginseng* which is 156,318 bp (GenBank Accession number: KJ566590). The length of IR regions is 26,126 bp each, 55 bp longer. The LSC region is 86,111 bp, 5 bp longer; and SSC is 18,024 bp, 46 bp shorter. There are 79 protein-coding genes, 30 tRNA genes, and 4 rRNA genes (Figure 1, Additional file 1: Table S1). The total G + C content of the whole chloroplast genome is 38.08%. The IRa/LSC, LSC/IRb and IRb/SSC junctions are identical to the chloroplast genome of *P. ginseng*, but the SSC/IRa junction (*ycf1*) of *P. notoginseng* is 8 bp shorter.

**Figure 2:**
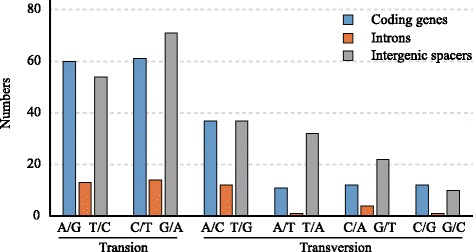
**The patterns of nucleotide substitutions among the two** ***Panax*** **chloroplast genomes.** The patterns were divided into 6 types as indicated by the six non-strand-specific base-substitution types (i.e., numbers of considered G to A and C to T sites for each respective set of associated mutation types). The chloroplast genome of *P. notoginseng* was used as a standard.

A comparison of the entire chloroplast genome sequences of *P. notoginseng* and *P. ginseng* revealed 464 nucleotide substitutions, including 273 transitions (Ts) and 191 transversions (Tv) (Figure 2). Of these substitutions, 193 events were in the coding regions, 45 in the introns and 226 in the intergenic spacers. The patterns among the three regions were similar. The proportion of Ts was much higher than that of Tv in all regions, indicating a bias in favor of transitions. This bias was even more pronounced in the coding region, in which the Ts/Tv was 1.68, whereas the Ts/Tv in the introns and intergenic spacers was 1.50 and 1.24, respectively. Among the 79 genes, 23 genes had non-synonymous substitutions.

In total, 156 indels were detected in the chloroplast genomes of two *Panax* species (Additional file 2: Table S2), 84 insertions and 72 deletions in *P. notoginseng* or 84 deletions and 72 insertions in *P. ginseng*. Most of the indels (63.06%) were single nucleotide differences. Indels longer than 10 bp occurred 16 times. The longest indel (34 bp) was in the spacer between *rps16* and *trnQ*. The majority of the indels occurred in the non-coding regions with two exceptions, a 15 bp insertion and an 18 bp insertion in the *ycf2* gene of *P. notoginseng*.

Three short inversions were observed in *ndhD-psaC, petB* intron and *trnM-atpE* between the two chloroplast genomes (Additional file 3: Table S3). All the inversions have hairpin structures, including the inversions and the inverted repeats. The inverted repeats formed the stem structures, and the inversions formed the loops. The lengths of inverted repeats were 3 bp, 44 bp, and 11 bp, and the lengths of the inversions were 19 bp, 18 bp, and 14 bp, respectively in the *ndhD-psaC, petB* intron and *trnM-atpE* regions.

### Variability throughout the chloroplast genomes

**Figure 3:**
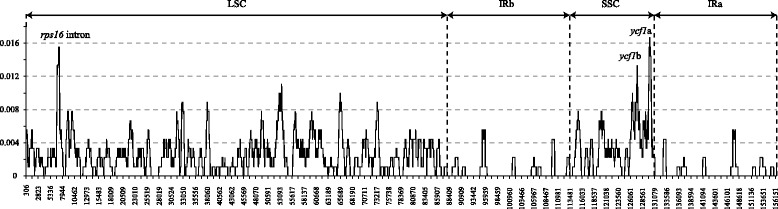
**Sliding window plots of nucleotide diversity (π) across the complete chloroplast genome of the two** ***Panax*** **species (window length: 600 bp, step size: 25 bp).** Y-axes: nucleotide diversity (π) of each window; X-axes: position of the midpoint of a window.

The variability throughout the chloroplast genomes was quantified using the average nucleotide diversity (π) (Figure 3). The average value of π is 0.00208. The IR regions exhibited lower variability than the LSC and SSC regions. There were three peaks which showed remarkably higher π values (>0.012). One is the intron of *rps16*, the other two are the coding regions of *ycf1* (*ycf1a and ycf1b*) (Figure 3). The variability of the three regions were tested together with the three conventional candidate barcodes (*matK, rbcL and trnH-psbA*) using 24 samples of all eight *Panax* species. The *ycf1a*, *ycf1b* and *trnH-psbA* showed nearly double the π values of the other three markers (Table 1).

**Table 1:** **Variability of the three new markers and universal chloroplast DNA barcode in** ***Panax***

**Markers**	**Length**	**Parsimony informative sites**		**Variable sites**		**Nucleotide diversity (π)**	**Indel**	**Number of haplotypes**
		**Numbers**	**%**	**Numbers**	**%**			
*rbcL*	637	15	2.35	15	2.35	0.0066	0	13
*matK*	818	30	3.67	30	3.67	0.0082	0	6
*trnH-psbA*	476	23	4.83	31	6.51	0.0140	6	11
*rps16*	848	23	2.71	26	3.07	0.0060	6	9
*ycf1a*	1094	116	10.60	113	10.33	0.0284	2	12
*ycf1b*	1186	69	5.81	74	6.24	0.0167	2	15

A barcoding analysis demonstrated that the *matK* and *trnH-psbA* can discriminate 62.50% of the samples. The percentages are 83.33% for *rps16* intron, 91.67% for *rbcL* and *ycf1a*, and 100% for *ycf1b* (Table 2).

**Table 2:** **The shortest length for a candidate barcode to reach the maximum discrimination success using genetic distance method**

**Markers**	**Maximum discrimination success (%)**	**Shortest length (bp)**	**Primer name**	**Primer sequence 5' to 3'**
*rbcL*	91.67	480	m-rbcLF	ACAAATTGACTTATTATACTCCTGA
			m-rbcLR	TCGTCTTTGGTAAAATCAAGTCCA
*matK*	62.5	90	m-matKF	CTTCTTGAACGAATCTATTTCTA
			m-matKR	CCATAAATTAACAAAGTAATATGT
*trnH-psbA*	62.5	50	m-HAF	TAATCTAGAATTTAGCTACTTCTTC
			m-HAR	CCTTGATCCACTTGGCTACATCC
*rps16*	83.33	280	m-rps16F	ATAGGAATGAAGGTGCTCTTG
			m-rps16R	ATCCTTCCAACAAAATGGCAGCA
*ycf1a*	91.67	60	m-ycf1aF	TTATTACCGAGTTGGAACAACA
			m-ycf1aR	TTGAGTACGCATAGAACCTTTGAT
*ycf1b*	100	110	m-ycf1bF	AAKCAAGAGACAACTTACCTTGA
			m-ycf1bR	GGATCAGATGCACAAAACCAAGGAA

### DNA mini-barcode for *Panax*

**Figure 4:**
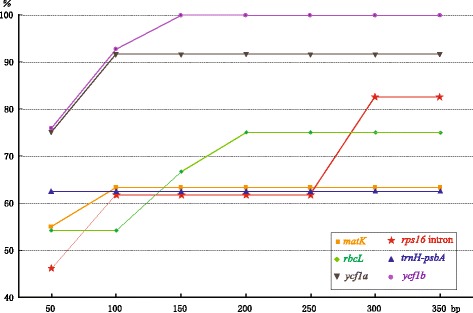
**Genetic distance-based discrimination power changes along with the increase of sequence lengths.** Pm: maximum percentage of samples discriminated.

Discrimination power, the maximum percentage of samples discriminated (Pm), varied with the increase of sequence lengths and among markers (Figure 4). The Pm of *trnH-psbA* never changes with the increase of sequence length. The Pm stabilized at 100 bp for *matK* and *ycf1a*, 150 bp for *ycf1b*, and 200 bp for *rbcL*. The Pm of *rps16* intron rose with the increase of sequence length (Figure 4). Since no change was observed on the Pm of *trnH-psbA*, the shortest mini-barcode is 60 bp of *ycf1a* with 91.67% of discrimination power (Table 2), whereas *ycf1b* needs 110 bp for a 100% of discrimination power. A pair of primer for the best mini-barcode of each marker was designed (Table 2). Powdered roots of *P. notoginseng* and steamed roots of *P. ginseng* purchased from market were used to test the mini-barcode (Additional file 4: Figure S1). Amplification and sequencing of *ycf1b* mini-barcode were successful, but amplification of the conventional *ycf1b* failed (Additional file 5: Figure S2).

## Discussion

Practically the length of a barcode becomes an issue of concern. Very subjectively we can classify a barcode according to the length, for example, micro-barcode within 100 bp, mini-barcode of 100–250 bp [2,11], conventional barcode of 250–1000 bp, super-barcode of 1000-6000 bp, and genome barcode using the whole genome. However, most applications of mini-barcodes are not necessarily to be the kind because a conventional barcode could be created by concatenating several mini-barcodes. Mini-barcodes were often used at the risk of lowering the resolution of taxa and consequently underestimated biodiversity. Mini-barcode has its potentials in situations that long fragments are impracticable or unnecessary for efficiency and economy considerations. Mini-barcodes of high resolutions are not easily found and that is why whole genomes are indispensible for development of mini-barcodes.

Chloroplast genome is endemic to plants. Chloroplast DNA barcodes bypass the DNA contamination from other organisms without chloroplasts, such as animals and fungi. Therefore, chloroplast DNA barcodes are of primary choices. Unfortunately, chloroplast genes usually evolve more slowly than nuclear genes [12] and the candidate barcodes such as *matK* and *rbcL* often have limited resolutions at species level [13,14]. However, there are some regions in the chloroplast genome which evolve much quickly and meet the criteria of being a DNA barcode. The strategy of searching the whole chloroplast genomes had been successfully applied to *Jacobaea* [15], *Oncidium* [16], *Parthenium* [17], and *Theobroma* [18]. Although some species are extremely closely related and no variations at the loci of *matK* and *rbcL*, for example, *Acorus americanus* v. s. *A. calemus* and *Oryza nivara* v. s. *O. sativa*, there are some differences at other loci [7]. Therefore, it is a reliable strategy to find the best chloroplast DNA mini-barcodes by searching the chloroplast genomes of congeners. Another advantage of chloroplast mini-barcode is that there is almost free of intra-populational variations and very low inter-populational variations. Sequence divergence is predominantly between species [19]. Species identification is for most cases more reliable.

Indels (gaps) are another kind of informative signals of potentially useful [20]. There are 157 indels along the two genomes of *Panax*. Indels are more useful at the lowest taxonomic level. Microsatellite markers are analogous to indels. It is often cumbersome in practice by using indel information. When gaps are coded as the fifth state of characters, they are very likely to be overweighted. To solve this problem, gaps are better coded manually. There is unlikely to have indels in the mini-barcodes of closely related species. However, chloroplast indels are likely to be another kind of DNA barcode for closely related species.

DNA mini-barcodes have so far been used for studying flora or fauna [3,4,9,11,21,22]. Such usages are often a compromise between resolution and experimental success. Consequently the mini-barcodes may underestimate the diversity of flora or fauna. However, DNA mini-barcodes are more suitable for ecologically and economically important taxa because it is more likely to find the best and taxa-specific mini-barcodes. Ginsengs are the most well-known herbal medicine in China. They have been extensively used for a long time. Substitution of expensive materials with similar but cheaper ones of congeners is reported occasionally. An effective and quick method for identifying the species of ginsengs is helpful for monitoring ginseng markers. We tested our mini-barcodes using materials purchased from market and they are proven applicable for such cases.

## Conclusions

In this study we provide a strategy for developing taxon-specific DNA mini-barcode without lowering discrimination power using the ginseng genus (*Panax*) as an example. The strategy by searching the whole genomes, identifying the most variable regions, shortening the focal regions for mini-barcodes are believed to be efficient in developing taxon-specific DNA mini-barcodes. The mini-barcodes for *Panax* were tested useful for identifying processed ginsengs from medicinal market.

## Methods

### Chloroplast genome sequencing

Leaves of *P. notoginseng* were collected from Wenshan, Yunnan province (Collection number: A8). The genomic DNA were extracted using modified CTAB (mCTAB) methods [23] and purified using the Wizard DNA Clean-Up System (Promega, Madison, WI, USA). The chloroplast genome was sequenced by using the short-range PCR method similar to Dong et al. [5]. *Panax*-specific primers (Additional file 6: Table S4) based on the chloroplast genome of *P. ginseng* [24] and some universal primers [5] were used to amplify and sequence the chloroplast genome of *P. notoginseng*. The chloroplast genome of ginseng served as a reference. The genome structure was confirmed by amplifying additional fragments spanning the LSC ↔ IRb, IRb ↔ SSC, SSC ↔ IRa, and IRa ↔ LSC [5].

### Genome annotation

The whole chloroplast genome was annotated using DOGMA [25] to identify the coding sequence, rRNA, and tRNA using the chloroplast/bacterial genetic code. The annotation of the tRNA genes was checked using tRNAscan-SE [26]. The genome map was generated using GenomeVx [27].

### Identification of the hypervariable regions

The chloroplast genome of *P. notoginseng* was aligned to the chloroplast genome of *P. ginseng* [24] using MAFFT [28] and then adjusted manually using Se-Al 2.0 [29]. To identify the highly variable regions within the chloroplast genomes, we calculated the nucleotide diversity using DnaSP ver. 5.0 [30] with a sliding window analysis. The window length was set to 600 bp with a step size of 25 bp.

### Plant material, PCR amplification and hypervariable region sequencing

All 8 *Panax* species were included in this study and each species was represented by at least two accessions (Additional file 7: Table S5). Medicinal materials (Additional file 4: Figure S1) were purchased from market to test the mini-barcodes designed in this study. The primers for amplifying the highly variable regions were designed using FastPCR (Additional file 8: Table S6). The primers for amplifying and sequencing the control markers of *rbcL, matK* and *trnH-psbA* were the same as Zuo et al. [10]. The PCR amplifications were performed in a final volume of 25 μL containing 1× PCR buffer (with Mg^2+^), 0.25 mmol/L each dNTP, 0.25 μmol/L each primer, 1.25 U Taq polymerase, and 20–30 ng DNA.

The PCR program started at 94°C for 4 min, followed by 34 cycles of 30 s at 94°C, 40 s at 52°C, and 1 min at 72°C, and ended with a final extension of 10 min at 72°C. The PCR products were checked by electrophoresis on a 1% agarose gel containing ethidium bromide and visualized using an ultraviolet transilluminator. Both of the strands were sequenced on ABI Prism 3730xl (Applied Biosystems, Foster City, U.S.A.) following the manufacturer’s protocols.

### DNA barcoding analysis

Distance is likely the most commonly used method for classifying DNA sequences. In this study, the distance method was used to analyze the barcoding performances of the newly identified highly variable regions. The function *nearNeighbour* of SPIDER was used for barcoding analysis [31]. Species discrimination was considered successful if the closest K2P distance for all of the individuals of a given species belonged to only one conspecific individual.

### DNA mini-barcode search using SPIDER

We used the sliding window function *slideAnalyses* of SPIDER [31] version 1.2-0 to find out the shortest informative windows. This function extracts all the passable windows of a chosen size in a DNA alignment and performs pairwise distance- (K2P) and NJ tree-based analyses of each window. In order to know the performances of markers with the increases of their sequence lengths, the changes of discrimination power, the maximum percentage of samples discriminated (Pm), at 50, 100, 150, 200, 250, 300, and 350 bp were depicted. In order to know the minimum length of a mini-barcode that performed as well as the full length, sliding window analyses were conducted. The starting length was set to 50 bp. The length was increased by 10 bp each round in the subsequent searches till the length of maximum discrimination power. The shortest length of a marker was considered the shortest mini-barcode of the marker.

### Availability of supporting data

The chloroplast genome of *P. notoginseng* has been submitted to GenBank (accession KJ566590). The data set supporting the results of this article is included in Additional file 7: Table S5 and available in the GenBank with accession number KM210094 – KM210203.

## Additional files

Additional file 1: Table S1. List of genes in the *Panax notoginseng* plastomes. Additional file 2: Table S2. The number and lengths of indel events in the chloroplast genome between the two *Panax* species. ^a^The chloroplast genome of *P. notoginseng* was used as a standard. Additional file 3: Table S3. Locations and sequences of three small inversions. Additional file 4: Figure S1. Photographs of processed ginsengs for sell in a medicine market. Additional file 5: Figure S2. PCR amplification profile of *ycf1b* mini-barcode and conventional *ycf1b* barcode of two processed ginsengs. A: powdered roots of *Panax notoginseng*; B: steamed roots of *P. ginseng*. Additional file 6: Table S4. List of primers used to amplify and sequence the genome of *Panax notoginseng* chloroplast genome. Additional file 7: Table S5. Vouchers and GenBank accessions for samples of *Panax*. Additional file 8: Table S6. Primers for PCR in the highly variable region.

## References

[1] Dong W, Cheng T, Li C, Xu C, Long P, Chen C, Zhou S (2014). Discriminating plants using the DNA barcode *rbcLb*: an appraisal based on a large dataset. Mol Ecol Resour.

[2] Meusnier I, Singer GA, Landry JF, Hickey DA, Hebert PD, Hajibabaei M (2008). A universal DNA mini-barcode for biodiversity analysis. BMC Genomics.

[3] Little DP (2014). A DNA mini-barcode for land plants. Mol Ecol Resour.

[4] Francoso E, Arias MC (2013). Cytochrome c oxidase I primers for corbiculate bees: DNA barcode and mini-barcode. Mol Ecol Resour.

[5] Dong W, Xu C, Cheng T, Lin K, Zhou S (2013). Sequencing angiosperm plastid genomes made easy: a complete set of universal primers and a case study on the phylogeny of Saxifragales. Genome Biol Evol.

[6] Dong W, Xu C, Cheng T, Zhou S (2013). **Complete chloroplast genome of*****Sedum sarmentosum*****and chloroplast genome evolution in Saxifragales**. PLoS ONE.

[7] Dong W, Liu J, Yu J, Wang L, Zhou S (2012). Highly variable chloroplast markers for evaluating plant phylogeny at low taxonomic levels and for DNA barcoding. PLoS ONE.

[8] Little DP, Jeanson ML (2013). DNA barcode authentication of saw palmetto herbal dietary supplements. Sci Rep.

[9] Shokralla S, Zhou X, Janzen DH, Hallwachs W, Landry JF, Jacobus LM, Hajibabaei M (2011). Pyrosequencing for mini-barcoding of fresh and old museum specimens. PLoS ONE.

[10] Zuo YJ, Chen ZJ, Kondo K, Funamoto T, Wen J, Zhou SL (2011). DNA barcoding of *Panax* species. Planta Med.

[11] Hajibabaei M, Smith MA, Janzen DH, Rodriguez JJ, Whitfield JB, Hebert PDN (2006). A minimalist barcode can identify a specimen whose DNA is degraded. Mol Ecol Notes.

[12] Drouin G, Daoud H, Xia J (2008). Relative rates of synonymous substitutions in the mitochondrial, chloroplast and nuclear genomes of seed plants. Mol Phylogenet Evol.

[13] Zhang CY, Wang FY, Yan HF, Hao G, Hu CM, Ge XJ (2012). Testing DNA barcoding in closely related groups of *Lysimachia* L. (Myrsinaceae). Mol Ecol Resour.

[14] Clement WL, Donoghue MJ (2012). Barcoding success as a function of phylogenetic relatedness in *Viburnum*, a clade of woody angiosperms. BMC Evol Biol.

[15] Doorduin L, Gravendeel B, Lammers Y, Ariyurek Y, Chin AWT, Vrieling K (2011). The complete chloroplast genome of 17 individuals of pest species *Jacobaea vulgaris*: SNPs, microsatellites and barcoding markers for population and phylogenetic studies. DNA Res.

[16] Wu FH, Chan MT, Liao DC, Hsu CT, Lee YW, Daniell H, Duvall MR, Lin CS (2010). Complete chloroplast genome of *Oncidium* Gower Ramsey and evaluation of molecular markers for identification and breeding in Oncidiinae. BMC Plant Biol.

[17] Kumar S, Hahn F, McMahan C, Cornish K, Whalen M (2009). Comparative analysis of the complete sequence of the plastid genome of *Parthenium argentatum* and identification of DNA barcodes to differentiate *Parthenium* species and lines. BMC Plant Biol.

[18] Kane N, Sveinsson S, Dempewolf H, Yang JY, Zhang D, Engels JM, Cronk Q (2012). **Ultra-barcoding in cacao (*****Theobroma*****spp.; Malvaceae) using whole chloroplast genomes and nuclear ribosomal DNA**. Am J Bot.

[19] Zhou S, Dong W, Chen X, Zhang X, Wen J, Schneider H (2014). **How many species of bracken (*****Pteridium*****) are there? Assessing the Chinese brackens using molecular evidence**. Taxon.

[20] Ochoterena H (2009). Homology in coding and non-coding DNA sequences: a parsimony perspective. Plant Syst Evol.

[21] Bhattacharjee MJ, Ghosh SK (2013). Design of mini-barcode for catfishes for assessment of archival biodiversity. Mol Ecol Resour.

[22] Arif IA, Khan HA, Al Sadoon M, Shobrak M (2011). Limited efficiency of universal mini-barcode primers for DNA amplification from desert reptiles, birds and mammals. Gen Mol Res.

[23] Li J, Wang S, Jing Y, Wang L, Zhou S (2013). A modified CTAB protocol for plant DNA extraction. Chinese Bulletin of Botany.

[24] Kim KJ, Lee HL (2004). Complete chloroplast genome sequences from Korean ginseng (*Panax schinseng Nees*) and comparative analysis of sequence evolution among 17 vascular plants. DNA Res.

[25] Wyman SK, Jansen RK, Boore JL (2004). Automatic annotation of organellar genomes with DOGMA. Bioinformatics.

[26] Schattner P, Brooks AN, Lowe TM (2005). The tRNAscan-SE, snoscan and snoGPS web servers for the detection of tRNAs and snoRNAs. Nucleic Acids Res.

[27] Conant GC, Wolfe KH (2008). GenomeVx: simple web-based creation of editable circular chromosome maps. Bioinformatics.

[28] Katoh K, Toh H (2010). Parallelization of the MAFFT multiple sequence alignment program. Bioinformatics.

[29] Rambaut A: **Se-Al: sequence alignment editor. version 2.0.** Oxford: University of Oxford, Department of Zoology; 1996.

[30] Librado P, Rozas J (2009). DnaSP v5: a software for comprehensive analysis of DNA polymorphism data. Bioinformatics.

[31] Brown SD, Collins RA, Boyer S, Lefort MC, Malumbres-Olarte J, Vink CJ, Cruickshank RH (2012). Spider: an R package for the analysis of species identity and evolution, with particular reference to DNA barcoding. Mol Ecol Resour.

