# Supplementary figures and images for: A chloroplast genomic strategy for designing taxon specific DNA mini-barcodes: a case study on ginsengs
Source: BMC Genet. 2014 Dec 20;15:138. doi: 10.1186/s12863-014-0138-z (PMC4293818; doi:10.1186/s12863-014-0138-z)

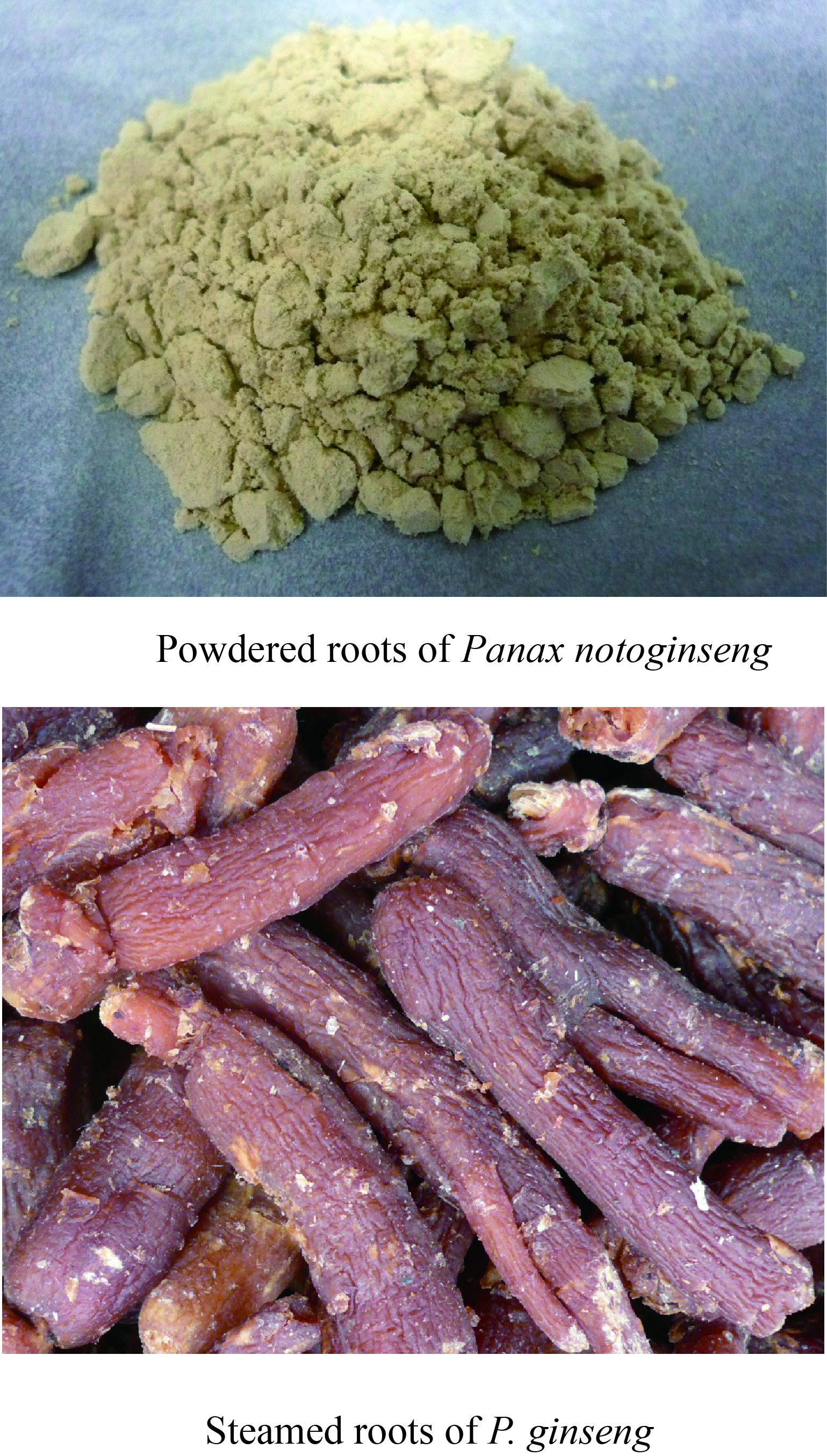

Supplement: Additional file 4: Figure S1. — Photographs of processed ginsengs for sell in a medicine market. [file 12863_2014_138_MOESM4_ESM.jpeg]

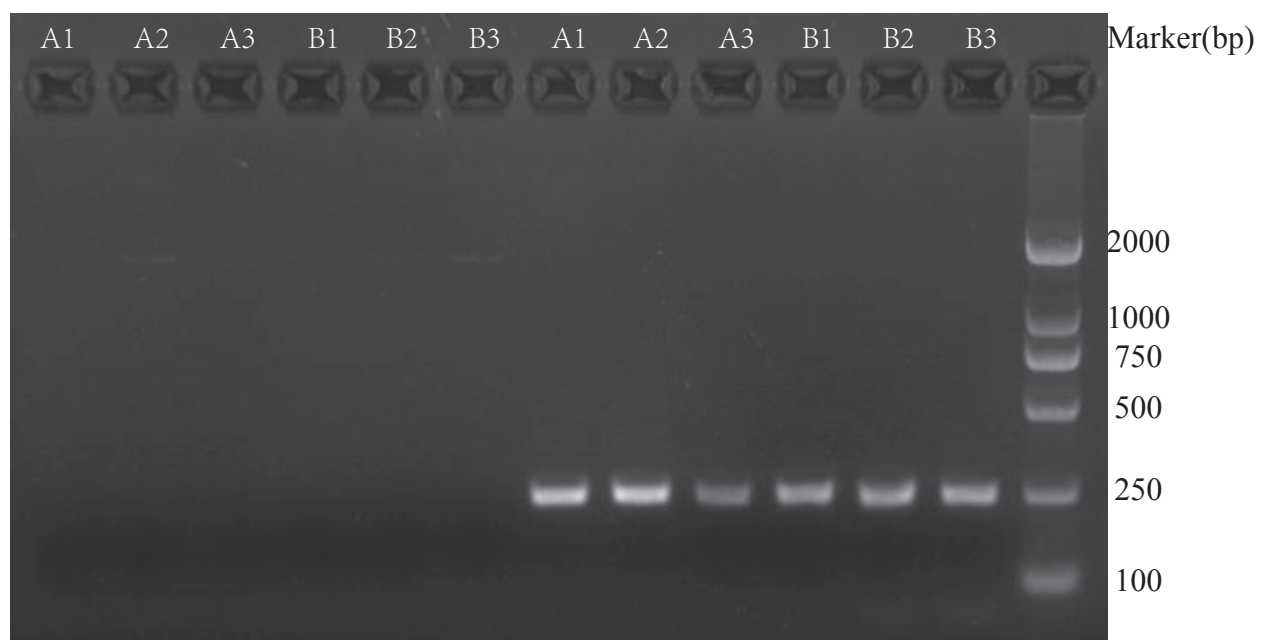

Supplement: Additional file 5: Figure S2. — PCR amplification profile of ycf1b mini-barcode and conventional ycf1b barcode of two processed ginsengs. A: powdered roots of Panax notoginseng; B: steamed roots of P. ginseng. [file 12863_2014_138_MOESM5_ESM.pdf]
